# Supplementary material for: Assessing the risk of concurrent mycoplasma pneumoniae pneumonia in children with tracheobronchial tuberculosis: retrospective study
Source: PeerJ. 2024 Mar 26;12:e17164. doi: 10.7717/peerj.17164 (PMC10979740; doi:10.7717/peerj.17164)
Supplement: Supplemental Information 1 [file peerj-12-17164-s001.docx]

**研究方案**

（版本号：2415，日期：2023.01.48）

项目名称：Construction of risk model pediatric tracheobronchial tuberculosis combined with Mycoplasma pneumoniae pneumonia-a retrospective study

申办单位： 南华大学衡阳医学院附属长沙中心医院

承担科室： 儿科

主要研究者： 谢齐放

参与单位：（若无请删除）

**研究者声明及方案签字页**

本人作为该研究项目的主要负责人，将遵循卫生部《涉及人的生命科学和医学研究伦理审查办法》（2023）、WMA《赫尔辛基宣言》（2013）和CIOMS《人体生物医学研究国际道德指南》（2002）和GCP的伦理原则，在药物临床试验质量管理规范指导下，使用伦理委员会批准的方案，根据本方案要求进行研究，以保证研究的科学性并保护受试者的健康与权利。

姓 名：________________

签 名：________________

日 期：________________

**方案摘要**

| 项目名称 | Construction of risk model pediatric tracheobronchial tuberculosis combined with Mycoplasma pneumoniae pneumonia-a retrospective study |
| --- | --- |
| 版本号/版本日期 |  |
| 申办及参与单位 |  |
| 主要研究者 | 谢齐放 |
| 研究性质 | 回顾性研究 |
| 研究目的 | 本研究旨在通过机器学习方法，如随机森林、LASSO、决策树等，对小儿气管支气管结核与肺炎支原体肺炎的合并症的特征进行筛选，进而利用logistics回归构建一个风险模型。该模型可以帮助医生更准确地预测和识别这种合并症，从而为患者提供更为精确和个性化的治疗方案。 |
| 样本量 | 小儿气管支气管结核与肺炎支原体肺炎的发生率为5%。，根据样本量计算公式，$\text{n}=\frac{\text{Z}^{2}\times p\times(1-p)}{E^{2}}$，其中：n 是所需的样本量，Z 是对应于给定的α的z值（在α=0.05时，双侧检验的Z值为1.96），p 是预期的发生率（0.05或5%），E 是可接受的误差范围（这里我们假设为0.05），计算结果约等于384例，考虑到10%的流失率，我们需要增加相应的样本量，这项研究应该计划纳入428例。但是样本量根据研究的中期结果进行调整。 |
| 研究对象 | 从2019年2月至2023年4月，收集的本院确诊为儿童气管支气管结核(TBTB)合并肺炎支原体肺炎(MPP)的患儿资料。同期确诊为大叶性支原体肺炎的患儿作为对照组。 |
| 研究方法 | 采用回顾性分析方法。数据收集涉及临床表现、实验室检查、影像学资料等。利用随机森林、LASSO、决策树筛选特征。再使用logistics回归构建风险模型。 |
| 纳入标准 | 1.在2019年2月至2023年4月期间，被本院确诊为儿童气管支气管结核(TBTB)合并肺炎支原体肺炎(MPP)的患儿。2.同期被确诊为大叶性支原体肺炎的患儿。3.临床资料完整。4.在本次研究前均为接受过其他治疗的患儿。 |
| 排除标准 | 1.诊断不明确或有争议的病例。2.在研究期间，接受了其他治疗或有其他合并症的患儿。3.排除合并有先天性心脏病、先天性免疫缺陷病等严重疾病的病例。 |
| 试验结束标准 |  |
| 脱落/剔除标准 |  |
| 提前退出标准 |  |
| 给药方案 |  |
| 主要疗效指标 |  |
| 次要疗效指标 |  |
| 安全性指标 |  |
| 研究进展计划 | 时间：2022年5月上旬至中旬  主要活动：进行初步的文献回顾，确定研究领域内的缺口，选择合适的研究题目。  理解论文题目和制定文献回顾计划：  时间：2022年6月上旬至下旬  主要活动：深入探讨题目的内涵，拟定查阅文献的计划，确定主要的研究问题和假设。  查阅文献并初步整合内容：  时间：2022年7月至8月上旬  主要活动：系统地搜索和查阅相关文献，整合研究的背景和意义。  预备知识研究与论文写作方法掌握：  时间：2022年8月下旬至9月上旬  主要活动：研究论文中涉及的关键理论和技术，了解并掌握论文的写作规范和方法。  深入研究与数据收集：  时间：2022年9月下旬至2023年1月中旬  主要活动：根据研究计划进行深入研究，完成实验或数据分析。  完成研究与论文初稿的撰写：  时间：2023年2月至5月  主要活动：根据研究成果，整理并初步完成论文草稿。  论文校对、修改和完善：  时间：2023年6月至8月  主要活动：对论文进行详尽的校对，完善格式和内容，确保达到期刊的标准。  选择合适的期刊并投稿：  时间：2023年10月  主要活动：基于论文的内容和领域，选择合适的学术期刊进行投稿。 |
| 统计学分析方法 | 数据处理：使用SPSS26.0进行初始的数据处理，涵盖了数据清洗、数据插补和随机分组。决策树和随机森林分析：通过SPSSAU平台进行。决策树用于识别数据中的主要分裂点，而随机森林模型用于评估每个变量的重要性并提高预测精度。使用R进行的高级统计分析：LASSO模型构建：使用"glmnet"包构建LASSO回归模型，帮助选择特征。Nomogram绘图：使用"rms"包绘制预测模型的图形表示。DCA和ROC绘图：使用"rmda"包进行DCA绘图，使用"rocr"包进行ROC曲线绘图。校准曲线和C-index计算：使用"rms"包绘制校准曲线并计算C-index。其他统计方法：正态分布的测量数据由均数±标准差（mean ± SD）描述，并通过t-test进行比较。对于独立样本，使用t-test比较组别。使用χ2检验比较计数数据，用百分比（%）表示。所有统计测试均为双侧检验，P值小于0.05被视为具有统计学意义。 |
| 研究成果发表形式 | 研究成果以论著发表形式，在国内外期刊上发表论文2-4篇（其中SCI 1篇） |

**一、研究背景**

**二、研究目的**

1. 主要目的
   本研究旨在通过机器学习方法，如随机森林、LASSO、决策树等，对小儿气管支气管结核与肺炎支原体肺炎的合并症的特征进行筛选，进而利用logistics回归构建一个风险模型。该模型可以帮助医生更准确地预测和识别这种合并症，从而为患者提供更为精确和个性化的治疗方案。
2. 次要目的
3. 探索性目的

**三、试验依据**

1. 研究前期的动物实验及文献基础
   a. 确定研究领域与话题：初步确定研究的方向和主题，确定关键词，以便进行文献搜索。b. 文献数据库的选择和搜索：使用PubMed, Web of Science, Scopus等科学数据库进行系统性文献搜索。筛选与评估：通过标题、摘要和关键词筛选相关性强的文献。阅读全文，评估文献的质量和相关性。d. 文献综述：整理已阅读文献的关键信息，如实验设计、结果、结论等。形成文献综述草稿，为研究提供理论基础。目前缺少关于小儿气管支气管结核与肺炎支原体肺炎的风险模型。因此，我们想建立符合本院要求的风险模型用于评估与预测患儿病情。
2. 受试者选择依据
   赫爽宇^[1]^和陈焱^[2]^的研究的研究文献我们制定那患者的纳入排除标准。
3. 剂量选择/给药方案/剂量调整依据
4. 终点选择依据
5. 风险及获益依据

**四、研究内容**

1. 试验人群
   从2019年2月至2023年4月，收集的本院确诊为儿童气管支气管结核(TBTB)合并肺炎支原体肺炎(MPP)的患儿资料。同期确诊为大叶性支原体肺炎的患儿作为对照组。
2. 样本量计算
   小儿气管支气管结核与肺炎支原体肺炎的发生率为5%。，根据样本量计算公式，，其中：n 是所需的样本量，Z 是对应于给定的α的z值（在α=0.05时，双侧检验的Z值为1.96），p 是预期的发生率（0.05或5%），E 是可接受的误差范围（这里我们假设为0.05），计算结果约等于384例，考虑到10%的流失率，我们需要增加相应的样本量，这项研究应该计划纳入428例。但是样本量根据研究的中期结果进行调整。
3. 具体研究内容
   通过机器学习方法，如随机森林、LASSO、决策树等，对小儿气管支气管结核与肺炎支原体肺炎的合并症的特征进行筛选，进而利用logistics回归构建一个风险模型。该模型可以帮助医生更准确地预测和识别这种合并症，从而为患者提供更为精确和个性化的治疗方案。

**五、研究方法**

1. 入组标准

（1）诊断标准：：参考中华人民共和国卫生行业标准-肺结核诊断（WS288-2017）[7]中的气管、支气管结核诊断标准，行纤维支气管镜检查直接观察到气管和支气管病变，活检病理、分泌物的结核分枝杆菌涂片、培养、核酸检测任一结果呈阳性，诊断为 TBTB。血清 MP-IgM、血清 MP-DNA 及肺泡灌洗液MP-DNA三者中两项为阳性者，临床上有肺炎表现和/或影像学改变，诊断为MPP。发热,常为高热

咳嗽、咳痰,可能有胸痛急性起病,病程一般在4周以内肺部物理检查可听到啰音或胸膜摩擦音。影像学表现:X线或CT显示单侧肺部肺野浸润影，占据一个或多个肺段或肺叶，可有胸膜反应。痰液检查:

痰液细菌培养阳性，革兰氏染色可见致病菌诊断为大叶性支原体肺炎。

（2）入选标准：1.在2019年2月至2023年4月期间，被本院确诊为儿童气管支气管结核(TBTB)合并肺炎支原体肺炎(MPP)的患儿。2.同期被确诊为大叶性支原体肺炎的患儿。3.临床资料完整。4.在本次研究前均为接受过其他治疗的患儿。

（3）排除标准：1.诊断不明确或有争议的病例。2.在研究期间，接受了其他治疗或有其他合并症的患儿。3.排除合并有先天性心脏病、先天性免疫缺陷病等严重疾病的病例。

1. 受试者分组
2. 试验治疗

（1）剂量选择/调整

（2）给药时间

（3）试验设盲/揭盲

（4）合并用药的标准

（5）补救药物与支持治疗（发生与研究相关SAE时必要的治疗措施）

1. 受试者提前退出/终止试验标准

**六、试验程序**

1. 受试者管理

（1）受试者的招募方式
我们进行的是一项回顾性研究，只需要收集患者病历进行分析

（2）知情同意过程/免知情同意书说明
本人将收集2019年02月212份临床数据，进行“构建儿童气管支气管结核合并肺炎支原体肺炎的风险模型-回顾性研究”研究。基于目前所有患者全部出院原因，故无法获取患者知情同意。

（3）核对入排标准

（4）检查病史及合并用药记录

（5）筛选编号的分配

（6）治疗/随机分组编号的分配

（7）试验依从性管理

1. 安全性评价程序（不良事件的评估、检测及报告）
2. 风险控制及管理程序
3. 疗效测量程序
4. 中止/退出程序
5. 设盲/揭盲程序
6. 访视要求

（1）筛选期

（2）治疗期

（3）治疗后的访视（安全性随访访视、随访访视、生存随访）

**七、试验的开始与结束**

**八、提前终止试验的临床标准**

**九、数据安全及监察计划**

1、医院信息系统具备完善的权限管理，本人将严格遵守医院管理要求；

2、研究相关的纸质资料于研究结束后5年销毁，或根据医院管理规定，统一由医院档案室进行管理；

3、本研究使用的临床数据所产生的数据均进行了匿名化处理，若发布研究结果，患者的身份信息仍将予以保密；

1. 数据管理方法概述
2. 不良事件和严重不良事件的报告和收集
3. 医疗安全措施
4. 与伦理委员会、上级药监部门的沟通
5. 数据的内部分析计划
6. 数据安全与监察报告递交给伦理委员会的频率

**十、伦理原则和相关法规的遵从性**

本研究将遵循现行赫尔辛基宣言和中国有关研究规范、法规进行。研究开始前报伦理委员会审核批准后实施。本方案在执行过程中，若需对本方案进行修订，则修订的研究方案再次报伦理委员会审批。

**十一、统计分析计划**

**深入研究与数据收集2022年9月下旬至2023年1月中旬，根据研究计划进行深入研究，完成实验或数据分析。**

**十二、数据、资料的保存和保密**

本研究将收集患者诊疗过程中的临床资料资料，受试者的个人信息资料将保存于医院，对外公开的数据资料以匿名化方式处理，不会涉及患者的姓名、性别等信息，确保患者隐私得到充分保护。为核实研究过程规范性和数据的真实性，研究者、研究监管部门人员和伦理委员会可以查阅受试者的原始资料。

**十四、研究成果的发表形式**

研究成果以论著发表形式，在国内外期刊上发表论文2-4篇（其中SCI 1篇）

**十五、参考文献**[1]赫爽宇. 儿童气管支气管结核合并肺炎支原体肺炎的临床特点及危险因素分析[D].中国医科大学,2022.DOI:10.27652/d.cnki.gzyku.2022.000953.
[2]陈焱. 肺结核合并气管支气管结核的风险预测模型的建立[D].重庆医科大学,2021.DOI:10.27674/d.cnki.gcyku.2021.001115.
