# Supplementary material for: Assessing the risk of concurrent mycoplasma pneumoniae pneumonia in children with tracheobronchial tuberculosis: retrospective study
Source: PeerJ. 2024 Mar 26;12:e17164. doi: 10.7717/peerj.17164 (PMC10979740; doi:10.7717/peerj.17164)
Supplement: Supplemental Information 2 [file peerj-12-17164-s002.docx]

**Research Proposal**

(version number: 2415, date: 2023.01.48)

Project title: Construction of risk model pediatric tracheobronchial tuberculosis combined with Mycoplasma pneumoniae pneumonia-a retrospective study

Sponsor: Changsha Central Hospital, Hengyang Medical College, South China University

Department: Paediatrics

Principal Investigator: Xie Qifang

Participating units: (delete if none)

**Researcher's statement and programme signature page**

As the main person in charge of this research project, I will follow the ethical principles of the Ministry of Health's Measures for Ethical Review of Life Science and Medical Research Involving Human Beings (2023), the WMA Declaration of Helsinki (2013) and the CIOMS International Ethical Guidelines for Biomedical Research on Human Beings (2002) and GCP, and will use the Ethics Committee, guided by the Code of Practice for Quality Management of Clinical Trials of Drugs, to approved protocol and conducted the study in accordance with the requirements of this protocol to ensure the scientific validity of the study and to protect the health and rights of the subjects.

Last name: ________________

Signature: ________________

Date: ________________

**Summary of the programme**

| Project name | Construction of risk model pediatric tracheobronchial tuberculosis combined with Mycoplasma pneumoniae pneumonia-a retrospective study |
| --- | --- |
| Version number/version date |  |
| Bidding and participating organisations |  |
| Principal Investigator | Xie Qifang (1930-), Chinese-American physicist, astronomer and mathematician |
| Nature of research | A retrospective study |
| research purpose | The aim of this study is to screen the features of the comorbidity of paediatric tracheobronchial tuberculosis with Mycoplasma pneumoniae pneumonia by machine learning methods such as Random Forest, LASSO, and Decision Tree, and then construct a risk model using logistics regression. The model can help physicians to predict and identify this comorbidity more accurately and thus provide more precise and personalised treatment plans for patients. |
| sample size | The incidence of paediatric tracheobronchial tuberculosis with Mycoplasma pneumoniae pneumonia was 5%. , according to the sample size formula,$\text{n}=\frac{\text{Z}^{2}\times p\times(1-p)}{E^{2}}$ , where: n is the required sample size, Z is the z-value corresponding to a given α (at α = 0.05, the z-value for a two-sided test is 1.96), p is the expected incidence (0.05 or 5%), and E is the acceptable margin of error (here, we have assumed 0.05), which is calculated to equal approximately 384 cases, and given the 10% attrition rate, we need to increase the sample size accordingly, and this study should plan to include 428 cases. However, the sample size will be adjusted according to the interim results of the study. |
| research target | Data collected from February 2019 to April 2023 on children diagnosed with childhood tracheobronchial tuberculosis (TBTB) combined with Mycoplasma pneumoniae pneumonia (MPP) in our hospital. Children diagnosed with Mycoplasma grandis pneumonia during the same period served as a control group. |
| Research methodology | A retrospective analysis method was used. Data collection involved clinical presentation, laboratory tests, and imaging data. Random forest, LASSO, decision tree were used to screen the features. Then logistic regression was used to construct the risk model. |
| Inclusion criteria | 1. children diagnosed with childhood tracheobronchial tuberculosis (TBTB) combined with Mycoplasma pneumoniae pneumonia (MPP) at our institution between February 2019 and April 2023.2. children diagnosed with Mycoplasma grandis pneumonia during the same period.3. complete clinical data.4. children who had received other treatments prior to the present study. |
| Exclusion criteria | 1. cases with unclear or controversial diagnosis. 2. children who received other treatments or had other comorbidities during the study period. 3. cases with comorbidities such as congenital heart disease, congenital immunodeficiency disease and other serious diseases were excluded. |
| End-of-test criteria |  |
| Shedding/rejection criteria |  |
| Early exit criteria |  |
| Drug delivery programme |  |
| Key efficacy indicators |  |
| Secondary efficacy indicators |  |
| Security Indicators |  |
| Research Progress Programme | Time: Early to mid-May 2022  Main activities: conduct an initial literature review, identify gaps in the research area and select appropriate research topics.  Understanding the dissertation topic and developing a plan for a literature review:  Time: Early to late June 2022  Main activities: in-depth exploration of the topic, development of a plan for reviewing the literature, and identification of the main research questions and hypotheses.  Review of literature and initial integration of content:  Time: July to early August 2022  Main activities: systematic search and review of relevant literature to integrate the context and significance of the study.  Preparatory knowledge research and essay writing methodology mastery:  Time: Late August to early September 2022  Main activities: research the key theories and techniques involved in the dissertation, and understand and master the norms and methods of dissertation writing.  In-depth research and data collection:  Duration: late September 2022 to mid-January 2023  Main activities: Conducting in-depth research and completing experiments or data analyses according to the research plan.  Completion of the first draft of the research and thesis:  Duration: February to May 2023  Main activity: Organise and complete a preliminary draft of the thesis based on the research results.  Thesis proofreading, revision and refinement:  Duration: June-August 2023  Main activities: Proofreading papers thoroughly, refining formatting and content to ensure that they meet the standards of the journal.  Select the appropriate journal and submit the manuscript:  Time: October 2023  Main activities: Selection of appropriate academic journals for submission based on the content and field of the paper. |
| Methods of statistical analyses | Data processing: initial data processing was carried out using SPSS 26.0, covering data cleaning, data interpolation and random grouping. Decision tree and random forest analyses: were carried out through the SPSSAU platform. Decision trees were used to identify major splits in the data, while random forest models were used to assess the significance of each variable and to improve prediction accuracy. Advanced statistical analyses using R: LASSO model construction: LASSO regression models were constructed using the 'glmnet' package to aid in feature selection.Nomogram plotting: Graphical representations of the predictive models were plotted using the 'rms' package.DCA and ROC plotting: DCA plots were performed using the 'rmda' package and ROC curves were plotted using the 'rocr' package. Calibration curves and C-index calculation: Calibration curves were plotted and C-index calculated using the "rms" package Other statistical methods: Normally distributed measurements were described by mean ± standard deviation (mean ± SD) and compared by t-test. For independent samples, groups were compared using t-test. Count data were compared using the χ2 test and expressed as percentages (%). All statistical tests were two-sided and a P value of less than 0.05 was considered statistically significant. |
| Forms of publication of research results | Research results in the form of treatises, 2-4 papers in domestic and international journals (including 1 SCI paper). |

**I. Background to the study**

**II. Purpose of the study**

1. Main Objective.
    The aim of this study is to screen the features of the comorbidity of paediatric tracheobronchial tuberculosis with Mycoplasma pneumoniae pneumonia by machine learning methods such as Random Forest, LASSO, and Decision Tree, and then construct a risk model using logistics regression. The model can help physicians to predict and identify this comorbidity more accurately and thus provide more precise and personalised treatment plans for patients.
2. secondary purpose
3. Exploratory purposes

**III. Test basis**

1. Animal experiments and literature base in the pre-study period
    a. Determination of research area and topic: Initially determine the direction and topic of the research and identify keywords for literature search. b. Selection and search of literature databases: Conduct a systematic literature search using scientific databases such as PubMed, Web of Science, Scopus, etc. c. Selection and search of literature databases: Conduct a systematic literature search using scientific databases such as PubMed, Web of Science, and Scopus. Screening and Evaluation: Screening the literature for relevance by title, abstract and keywords. Read the full text to assess the quality and relevance of the literature. d. Literature review: Collate key information from the read literature, such as experimental design, results, conclusions, etc. Form a draft literature review to provide a theoretical basis for the study. There is a lack of risk models on paediatric tracheobronchial tuberculosis and Mycoplasma pneumoniae pneumonia. Therefore, we would like to develop a risk model for assessment and prediction of children's conditions that meets the requirements of our institution.
2. Subject selection was based on
    The research literature of the studies of Shuangyu Heh^[1]^ and Yan Chen^[2]^ We developed the inclusion exclusion criteria for that patients.
3. Dose selection/administration regimen/basis for dose adjustment
4. Basis for endpoint selection
5. Risk and benefit basis

**IV. Content of the study**

1. Trial Population.
    Data collected from February 2019 to April 2023 on children diagnosed with childhood tracheobronchial tuberculosis (TBTB) combined with Mycoplasma pneumoniae pneumonia (MPP) in our hospital. Children diagnosed with Mycoplasma grandis pneumonia during the same period served as a control group.
2. Sample size calculation
    The incidence of paediatric tracheobronchial tuberculosis with Mycoplasma pneumoniae pneumonia was 5%. , according to the sample size calculation formula,, where: n is the required sample size, Z is the z-value corresponding to a given α (at α = 0.05, the z-value for a two-sided test is 1.96), p is the expected incidence (0.05 or 5%), and E is the acceptable margin of error (here we assume 0.05), which is calculated to equal approximately 384 cases, and given the 10% attrition rate, we would need to increase the sample size accordingly, and this study should plan to include 428 cases. However, the sample size will be adjusted according to the interim results of the study.
3. Specific research content
    The features of the comorbidity between paediatric tracheobronchial tuberculosis and Mycoplasma pneumoniae pneumonia are screened by machine learning methods such as Random Forest, LASSO, Decision Tree, etc., and then a risk model is constructed by using logistics regression. The model can help physicians to predict and identify this comorbidity more accurately and thus provide more precise and personalised treatment plans for patients.

**V. Research methodology**

1. Inclusion criteria

(1) Diagnostic criteria:: Referring to the diagnostic criteria for tracheal and bronchial tuberculosis in the health industry standard of the People's Republic of China - Diagnosis of pulmonary tuberculosis (WS288-2017) [7], tracheal and bronchial lesions were directly observed by fibreoptic bronchoscopy, and the diagnosis of TBTB was made with a positive result of biopsy pathology, Mycobacterium tuberculosis smears of secretion, culture, and nucleic acid testing. serum MP- IgM, serum MP-DNA, and alveolar lavage MP-DNA were positive, and clinical manifestations of pneumonia and/or imaging changes were made. IgM, serum MP-DNA, and alveolar lavage MP-DNA are positive, and the diagnosis of MPP is made if there are clinical signs of pneumonia and/or imaging changes. fever, often high.

Cough, sputum, may have chest painAcute onset, disease duration usually less than 4 weeksPhysical examination of the lungs may reveal rales or pleural friction. Imaging:X-ray or CT shows unilateral infiltrating shadows in the lung fields, occupying one or more lung segments or lobes, and pleural reaction may be present. Sputum Examination.

Positive sputum bacterial culture and Gram staining showed the causative organisms diagnosed as Mycoplasma hyopneumoniae lobarum.

(2) Inclusion criteria: 1. children diagnosed with childhood tracheobronchial tuberculosis (TBTB) combined with Mycoplasma pneumoniae pneumoniae (MPP) by our institution between February 2019 and April 2023. 2. children diagnosed with Mycoplasma grandis pneumonia during the same period. 3. complete clinical data. 4. all children who had received other treatments prior to the current study. 5. children who had received other treatments prior to the current study. 6. children who had received other treatments prior to the current study. 7. children who had received other treatments prior to the current study. 8. children who had received other treatments prior to the current study. 9. children who had been treated by our hospital.

(3) Exclusion criteria: 1. Cases with unclear or controversial diagnosis. 2. Children who received other treatments or had other comorbidities during the study period. 3. Excluding cases with comorbidities such as congenital heart disease, congenital immunodeficiency disease, and other serious diseases. 4.

1. Subject grouping
2. experimental treatment

(1) Dose selection/adjustment

(2) Time of administration

(3) Test blinding/blinding

(4) Criteria for Combined Use

(5) Remedial medications and supportive care (treatment necessary in the event of a research-related SAE)

1. Criteria for early subject withdrawal/termination of the trial

**VI. Test procedures**

1. Subject management

(1) How subjects were recruited
 We conducted a retrospective study that required only the collection of patient medical records for analysis

(2) Informed Consent Process / Waiver of Informed Consent Statement
 I will collect 212 clinical data on 02/2019 for the study "Constructing a risk model for tracheobronchial tuberculosis combined with Mycoplasma pneumoniae pneumonia in children - a retrospective study". Informed consent could not be obtained from the patients due to the fact that all patients are currently discharged from the hospital.

(3) Reconciliation of entry criteria

(4) Examination of medical history and records of co-medication

(5) Assignment of screening numbers

(6) Assignment of treatment/randomisation group numbers

(7) Trial Adherence Management

1. Safety evaluation procedures (assessment, detection and reporting of adverse events)
2. Risk control and management procedures
3. Efficacy measurement procedures
4. Aborting/exiting the programme
5. Blinding/unblinding procedures
6. Visiting requirements

(1) Screening period

(2) Treatment period

(3) Post-treatment visits (safety follow-up visits, follow-up visits, survival follow-up visits)

**VII. Beginning and end of the experiment**

**VIII. Clinical criteria for early termination of trials**

**IX. Data security and monitoring programmes**

1, the hospital information system with perfect rights management, I will strictly abide by the hospital management requirements;

2. Research-related paper materials will be destroyed 5 years after the end of the study, or will be managed uniformly by the hospital archives in accordance with hospital management regulations;

3. The data generated from the clinical data used in this study have been anonymised, and if the results of the study are published, the identity of the patients will remain confidential;

1. Overview of data management methodologies
2. Reporting and collection of adverse events and serious adverse events
3. Medical safety measures
4. Communication with ethics committees, higher drug regulatory authorities
5. Internal analysis plan for the data
6. Frequency of submission of data security and monitoring reports to the Ethics Committee

**x. ethical principles and compliance with relevant regulations**

The study will be conducted in accordance with the current Declaration of Helsinki and relevant Chinese research norms and regulations. The study will be submitted to the Ethics Committee for review and approval before commencement of the study. If there is a need to revise the protocol during the implementation of the study, the revised study protocol will be submitted to the Ethics Committee again for approval.

**XI. Statistical analysis plan**

**In-depth research and data collection Late September 2022 to mid-January 2023 In-depth research and completion of experiments or data analysis according to the research plan.**

**XII. Preservation and confidentiality of data and information**

This study will collect clinical data information during the patient's diagnosis and treatment, and the subjects' personal information data will be kept in the hospital, and the externally disclosed data information will be handled in an anonymised manner, without involving the patient's name, gender and other information, to ensure that the patient's privacy is fully protected. In order to verify the standardisation of the research process and the authenticity of the data, the researcher, the research supervisory authority and the ethics committee will have access to the original data of the subjects.

**XIV. Forms of publication of research results**

Research results in the form of treatises, 2-4 papers in domestic and international journals (including 1 SCI paper).

**XV. References** [1] He Shuangyu. Analysis of clinical characteristics and risk factors of tracheobronchial tuberculosis combined with Mycoplasma pneumoniae pneumonia in children[D]. China Medical University, 2022.DOI:10.27652/d.cnki.gzyku.2022.000953.
 [2] Yan Chen. Establishment of risk prediction model for pulmonary tuberculosis combined with tracheobronchial tuberculosis[D]. Chongqing Medical University,2021.DOI:10.2 7674/d.cnki.gcyku.2021.001115.
