# Supplementary material for: Assessing the risk of concurrent mycoplasma pneumoniae pneumonia in children with tracheobronchial tuberculosis: retrospective study
Source: PeerJ. 2024 Mar 26;12:e17164. doi: 10.7717/peerj.17164 (PMC10979740; doi:10.7717/peerj.17164)
Supplement: Supplemental Information 4 [file peerj-12-17164-s004.docx]

1.Is the research question and purpose clearly stated?

Yes

2. Have the variables used in the prediction model and their measurement methods been defined?

Yes

3. Have you clearly described the construction method and process of the prediction model?

Yes

4. Have estimates and explanations of sample size been provided?

Yes
5.sample selection, and data collection?

Yes

6. Did you explain the situation of missing data and provide a handling method?

Yes

7. Have you clearly described the statistical methods and algorithms of the prediction model?

Yes

8. Have you explained the selection and exclusion criteria for each variable in the model?

Yes

9.Has the distribution and absence of each variable in the model been reported?

No

10. Have the adaptability indicators of the prediction model, such as fit, correction, and discrimination, been reported?

Yes

11.Does it explain the stability and repeatability of the prediction model?

Yes

12.Has the bias and error of the prediction model been evaluated?

Yes

13. Have internal validation results of the prediction model been provided?

Yes

14. Have external validation results of the prediction model been provided?

Yes

15. Have you explained the potential application scenarios and audience groups of the prediction model?

Yes

16. Is an interpretability analysis of the predictive model provided?

Yes

17.Has the uncertainty of the prediction model been evaluated?

Yes

18.Did you explain the limitations and limitations of the predictive model?

Yes

19. Are performance indicators for the prediction model provided, such as sensitivity, specificity, and ROC curve?

Yes

20.Has the utility and economic cost-effectiveness of the prediction model been evaluated?

Yes

21.Has the effectiveness and clinical significance of the model been reported?

Yes

22. Have external application guidelines for the model been provided?

Yes

23. Is the source code and data for the prediction model provided?

Yes

24. Is registration and review information provided for the prediction model?

Yes

25. Has the derivation and update information of the prediction model been provided?

Yes
